# A dataset on the inventory of coniferous urban trees in the city of Orléans (France)

**DOI:** 10.1016/j.dib.2016.10.015

**Published:** 2016-10-24

**Authors:** J.-P. Rossi, V. Imbault, T. Lamant, J. Rousselet

**Affiliations:** a INRA, UMR CBGP, Campus International de Baillarguet, CS 30016, F-34988 Montferrier-sur-Lez Cedex, France; b INRA, UR633 Zoologie Forestière, 2163 avenue de la pomme de pin, Ardon, CS 40001, 45075 Orléans cedex 2, France; c ONF, CGAF, 2163 avenue de la pomme de pin, Ardon, CS 40001, 45075 Orléans cedex 2, France

**Keywords:** Urban trees, Trees outside forests

## Abstract

The dataset supplied in this article provides the spatial location and the species composition of urban trees belonging to three coniferous genera (*Pinus*, *Cedrus* and *Pseudotsuga*) inventoried in 5 districts of the city of Orléans (France). A total of 9321 trees were georeferenced. The most abundant species was the black pine *Pinus nigra* for which a total of 2420 trees were observed. Other common species were the scots pine *P. sylvestris*, the Douglas-fir *Pseudotsuga menziesii* and different species of the genus *Cedrus*. The data supplied in this article are related to “A citywide survey of the pine processionary moth *Thaumetopoea pityocampa* spatial distribution in Orléans (France)” by J.-P. Rossi, V. Imbault, T. Lamant, J. Rousselet,) [3].

**Specifications Table**

**Table:** 

Subject area	*Biology*
More specific subject area	*Urban forestry*
Type of data	*Table, shapefiles*
How data was acquired	*Field observation*
Data format	*Raw*
Experimental factors	*Field data: a set of 9321 urban trees were identified and georeferenced*
Experimental features	*Urban trees were identified and georeferenced during fieldwork carried out in 5 municipalities of the Orléans urban community*
Data source location	*The city of Orléans (France)*
Data accessibility	Data are available within this article

**Value of the data**

• Each tree was georeferenced and the dataset allows the statistical analysis of trees spatial distribution across the city.
• Eleven taxonomic units were recorded and the dataset thus allows the citywide analysis of species association.
• The dataset could be used in comparative studies of ornamental tree species composition and association.

## 1 Data

**Fig. 1:**
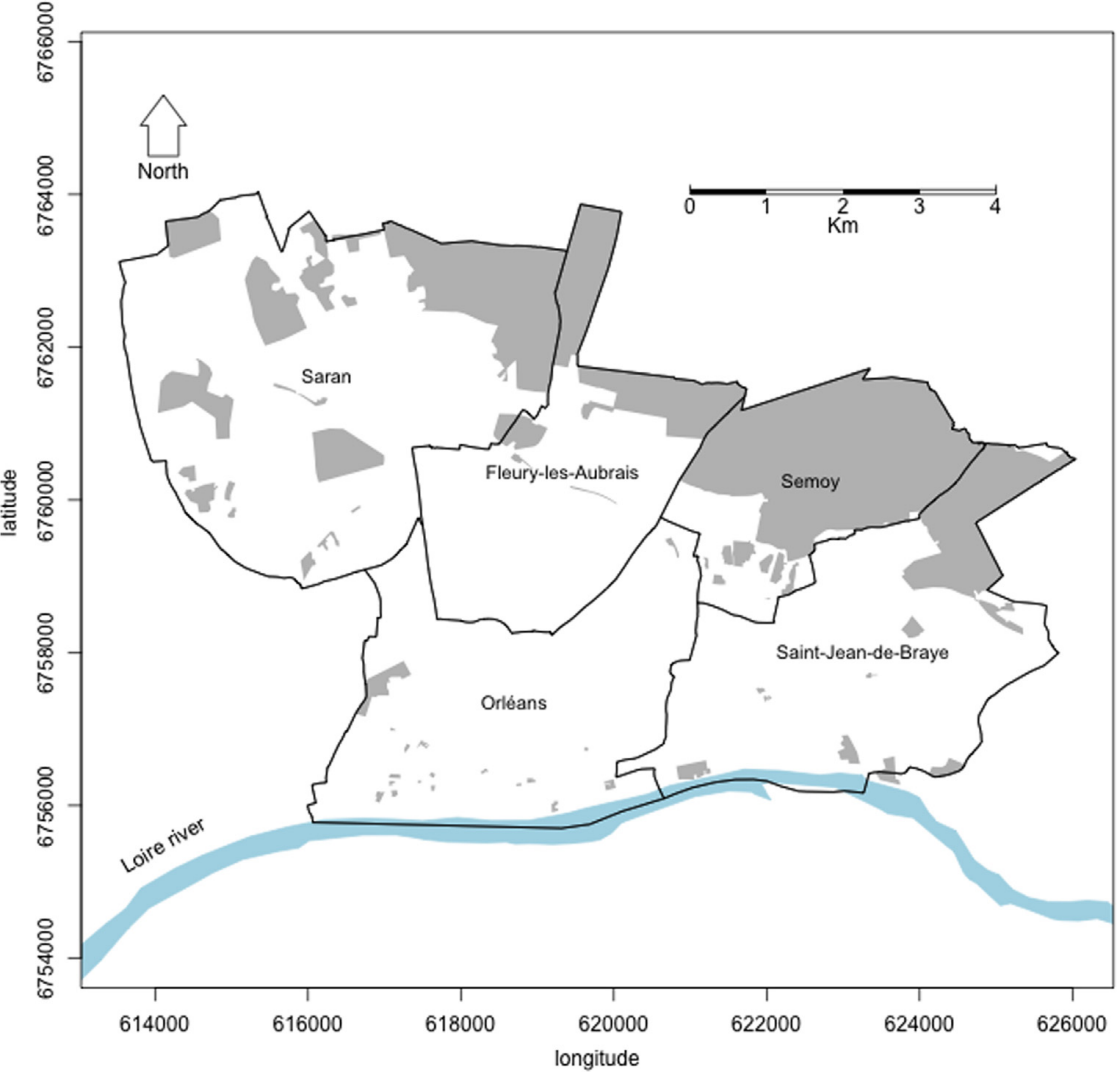
Study site comprising 5 districts of the Orléans agglomeration: Fleury-les-Aubrais, Orléans, Saint-Jean de Braye, Saran and Semoy. The district of Orléans extents both sides of the Loire River and our survey focused on the northern part. Shaded areas denote areas where urban trees were not inventoried (see text for details).

The dataset of this article provides an exhaustive inventory of the urban trees belonging to the genera *Pinus*, *Cedrus* and *Pseudotsuga* (Pinaceae) in 5 municipalities of the Orléans Val de Loire urban community (Fig. 1). The total inventoried surface was 4903 ha and included both public and private lands. A total of 9321 trees planted in parks, gardens and yards or along streets and roads and representing 11 coniferous taxonomic units (see below) were identified and georeferenced.

## 2 Experimental design, materials and methods

### 2.1 Study area

The survey was undertaken in 5 municipalities in the north of the Orléans Val de Loire agglomeration, namely Fleury-les-Aubrais, Orléans, Saint-Jean de Braye, Saran and Semoy (Fig. 1). Some areas were excluded from the inventory because access was impossible or forbidden (*e.g.* military settlements). Overall, un-sampled surfaces (shaded areas in Fig. 1) represented a total of 1580 ha corresponding to 24.3% of the survey area (6483 ha). The total inventoried surface was 4903 ha.

### 2.2 Field measurements

The dataset was collected as part of a project focused on the citywide spatial distribution of the pine processionary moth *Thaumetopoea pityocampa* Denis and Schiffermüller (Lepidoptera, Notodontidae) [4]. This species is a pine defoliator (genus *Pinus*) that occasionally feeds on other coniferous taxa such as Cedrus or *Pseudotsuga* under natural conditions [2]. Our inventory thus focused on these genera *i.e.* the genus *Pinus*, *Cedrus* and *Pseudotsuga* [4].

Street and garden trees were observed from the road and public land and their geographic coordinates were recorded using a GETAC PS236. Distant trees were mapped onto a georeferenced aerial photo using ArpentGIS mobile D3E Electronique. We used the EPSG projection 2154 (RGF93 / Lambert93). Details are given in [3].

A total of 9321 trees were georeferenced. The most common species were the black pine *P. nigra*, the scots pine *P. sylvestris*, the Douglas-fir *P. menziesii* and cedars (*C. atlantica/C. libani* and *C. deodara*). We observed 5 native European pine species (*P. mugo*, *P. nigra*, *P. pinaster*, *P. pinea*, and *P. sylvestris*), 2 North-American taxa (*Pinus subgenus Strobus* and *Pseudotsuga menziesii*) and 3 cedar species originating from North Africa (*Cedrus atlantica*, Atlas cedar), Near East (*C. libani*, Lebanon cedar) and Asia (*C. deodara*, Himalayan cedar) [1]. *C. atlantica* and *C. libani* were gathered in one unique taxonomic unit referred to as *C. atlantica/C. libani* because they are hardly discernable in the field.

In some cases, identification to the species level was impossible because trees were too distant. This corresponded to 16 individuals of genus *Cedrus* (referred to as *Cedrus spp.*), 13 individuals of genus *Pinus* (referred to as *Pinus spp.*) and 338 trees of genus *Pinus* belonging to the subgenus *Strobus*, section *Quinquefoliae*, subsection *Strobus* (referred to as *Pinus* subgenus *Strobus*).
